# Basophils and Mast Cells in COVID-19 Pathogenesis

**DOI:** 10.3390/cells10102754

**Published:** 2021-10-14

**Authors:** Giuseppe Murdaca, Mario Di Gioacchino, Monica Greco, Matteo Borro, Francesca Paladin, Claudia Petrarca, Sebastiano Gangemi

**Affiliations:** 1 Clinical Immunology Unit, Department of Internal Medicine, University of Genoa and Ospedale Policlinico San Martino, 16132 Genoa, Italy; giuseppe.murdaca@unige.it; 2 Center for Advanced Studies and Technology, G’ d’Annunzio University, 66100 Chieti, Italy; claudia.petrarca@unich.it; 3 Institute for Clinical Immunotherapy and Advanced Biological Treatments, 65100 Pescara, Italy; 4 Internal Medicine Department, San Paolo Hospital, 17100 Savona, Italy; monicagreco@gmail.com (M.G.); borromatteo@libero.it (M.B.); 5 Department of Internal Medicine, University of Genoa, Ospedale Policlinico San Martino IRCCS, 16132 Genoa, Italy; puell-a@hotmail.it; 6 Department of Medicine and Aging Sciences, G. d’Annunzio University, 66100 Chieti, Italy; 7 Department of Clinical and Experimental Medicine, School and Operative Unit of Allergy and Clinical Immunology, University of Messina, 98125 Messina, Italy; sebastiano.gangemi@unime.it

**Keywords:** basophils, mast cells, COVID-19, innate immune response, adaptive immune response

## Abstract

Basophils and mast cells are among the principal inducers of Th2 responses and have a crucial role in allergic and anti-parasitic protective immunity. Basophils can function as antigen-presenting cells that bind antigens on their surface and boost humoral immune responses, inducing Th2 cell differentiation. Their depletion results in lower humoral memory activation and greater infection susceptibility. Basophils seem to have an active role upon immune response to SARS-CoV-2. In fact, a coordinate adaptive immune response to SARS-CoV-2 is magnified by basophils. It has been observed that basophil amount is lower during acute disease with respect to the recovery phase and that the grade of this depletion is an important determinant of the antibody response to the virus. Moreover, mast cells, present in a great quantity in the nasal epithelial and lung cells, participate in the first immune response to SARS-CoV-2. Their activation results in a hyperinflammatory syndrome through the release of inflammatory molecules, participating to the “cytokine storm” and, in a longer period, inducing pulmonary fibrosis. The literature data suggest that basophil counts may be a useful prognostic tool for COVID-19, since their reduction is associated with a worse prognosis. Mast cells, on the other hand, represent a possible therapeutic target for reducing the airway inflammation characteristic of the hyperacute phase of the disease.

## 1. Introduction

The outbreak of severe acute respiratory syndrome coronavirus 2 (SARS-CoV-2), which was first reported in Wuhan in December 2019 and is responsible for coronavirus disease 2019 (COVID-19), rapidly spread throughout the world, causing an ongoing, highly contagious, pandemic emergency [1,2]. The Worldometer coronavirus (www.worldometers.info/coronavirus/ accessed date on 11 October 2021) recorded more than 238,760,310 COVID-19 cases, with 4860 million deaths in 222 countries and territories. Since the beginning, several attempts have been made to defeat the virus, starting from social behavioral strategies, such as social distancing and mask wearing, to pharmacological discoveries (i.e., monoclonal antibodies against S protein) and immunization protocols [3,4]. Indeed, several vaccine platforms have been studied, with some of them already having received regulatory agencies approval, thus including inactivated, live attenuated, and protein/adjuvant approaches and viral vectors and nucleic acids [5].

SARS-CoV-2 belongs to the beta-coronavirus genus of the family Coronaviridae, consisting of four different genera: alpha-coronavirus, beta-coronavirus, gamma-coronavirus, and delta-coronavirus (ICTV Virus Taxonomy: 2019 Release) [6]. Coronaviruses are characterized by the presence of an envelope and a single-stranded, positive-sense RNA genome 29–30 kb in size. They infect numerous animal species including humans and present high interspecies transfers thus being important zoonotic pathogens. In particular, bats and birds are considered the “natural reservoirs” for human coronavirus zoonotic infections, and all human coronavirus represents the result of a zoonotic transfer (“spillover”) from the animal reservoir, either directly or through an intermediate animal host [7]. Like other human coronaviruses, SARS-COV-2 is an enveloped single-stranded positive RNA (ssRNA) virus. The genome is packed by viral nucleocapsid (N) proteins as a large ribonucleoprotein (RNP) complex and further enclosed by an envelope membrane made of lipids and viral proteins S (surface or spike), M (membrane), and E (envelope) [6], thus forming a sort of “corona”.

The virus enters the target host cells through the S protein, which binds to the angiotensin-converting enzyme 2 (ACE2) receptor on the cell surface through the action of transmembrane protease serine (TMPRSS2) [8,9], which mediates the cleavage of the S protein itself and initiates the fusion of viral and host membranes. Both ACE2 and TMPRSS2 are expressed in many cell types, with particularly high prevalence in lungs and intestine epithelia and endothelial cells, allowing SARS-CoV-2 to target numerous vital organs. For the same reason, SARS-CoV-2 may thus induce a wide spectrum of symptoms [10]. As an RNA virus, SARS-CoV-2, after entering the host cell, replicates exclusively in the cytoplasm of infected cells, where the viral genome is first unpacked from bound viral N proteins by cellular proteases [6].

When inhaled, SARS-CoV-2 is recognized by pattern recognition receptors (PRRs), thus activating the recruitment of innate and adaptive immune system. Notably, IFNs type I amplify the inflammatory signal, thus promoting inflammatory cytokines, chemokines, and anti-viral enzyme production. At this stage, SARS-CoV-2 locally replicates, and patients are often asymptomatic, but still highly infectious [11]. As viral spread goes on, further cells are infected and viral peptides are presented on MHC I to CD8+ T cells [12,13], leading to clonal expansion of specific memory cells. Cytotoxic T cells are now activated to clear the virus thanks to perforin and granzyme production [14]. This scenario also includes innate cells co-adjuvate T cells such as macrophages, monocytes, and neutrophils. When the combined action of these players overwhelms the system balance, cytokine storm is induced, often leading to acute respiratory distress syndrome (ARDS) and multi-organ failure [15,16,17].

### Basophils and Mast Cells in Viral Infections

Granulocytes are white blood cells (WBCs) characterized by the presence of lobulated nuclei and secretory granules in their cytoplasm and mainly comprise neutrophils, basophils, and eosinophils. Activated human basophils and mast cells are characterized by the expression of the high affinity receptor for IgE (FcεRI), synthesizing histamine and the ability to produce preformed and synthesized de novo mediators. The expression of a wide range of pattern recognition receptors suggests that they play a role in various forms of innate host immunity, as well as new evidence regarding their regulatory abilities of adaptive host immunity [18,19]. Specifically, human basophils release proinflammatory mediators and a restricted profile of cytokines (IL-4 and IL-13) and chemokines (CXCL8/IL-8 and CCL3/MIP-1α), unlike human mast cells, which are instead capable of expressing a broad spectrum of cytokines and chemokines [20].

Basophils and mast cells play a fundamental role in different types of viral infections.

For example, in HIV infection, basophils and mast cells can capture HIV-1 and mediate viral transinfection of CD4+ T cells by the expression of a variety of attachment factors to the virus, such as type C lectins.

Furthermore, mast cells can recruit different types of cells, such as T cells, macrophages, dendritic cells, and neutrophils to the site of infection for the elimination of invading pathogens [19,21].

Given that basophils express high levels of the high affinity IgE receptor FcεRI, it has been shown that exposure of basophils to rhinovirus preferentially increase the response to IgE in atopic asthmatics by amplification of the IgE/TSLPR axis, thus prolonging the inflammatory processes following viral infection [22]. Several recent studies indicate that basophils also play a key role in the induction of Th2 inflammation, in particular in respiratory syncytial virus (RSV) infection, in which there is an accumulation of these granulocytes in the lung, with consequent release of IL-4 and inflammation of the parenchyma [23,24].

Mast cells (MCs) also play a key role as immune sentinels against various types of viral infections. These cells in fact not only express a variety of Toll-like receptors (TLR-3 and TLR-9) for recognition of virus-derived PAMP molecules, but also molecules such as RIG-I, NOD, and the mannose receptor (CD48) [25,26].

In some mouse studies conducted on H5N1 highly pathogenic avian influenza virus infection (H5N1-HPAIV), it was shown that the production of defective viral particles (DP) by MCs thanks to the presence of Argonaute 2 RNAase (AGO2) can interfere with virus replication and stimulate the innate immune response of host cells. Furthermore, in this type of viral infection, activated MCs have been shown to be responsible for histamine release when avian subtype H5N1 influenza A virus-sensitized mice lacking neutralizing antibodies are infected with a derived influenza virus, with an increased risk of a more severe flu-like illness [27,28].

The role of histamine as a mediator related to disease severity is also recognized in patients infected with the Dengue virus, in which MCs are responsible for the production of the T cell chemotactic agents RANTES, MIP-1α, and MIP-1β and therefore play a crucial role in the pathogenesis of this severe viral disease [29]. Brown et al. [30] also demonstrated how mast cells respond to dengue virus infection by producing type I interferons and chemokines including CCL4, CCL5, and CXCL10, in addition to TNF-α, responsible for inducing endothelial activation and consequent vascular losses, a hallmark of severe Dengue virus infection [31,32].

MCs also play protective roles against viral infections, which is what occurs for example in HSV-2 infections, in which the production by these cells of TNF-α and IL-6 determine a direct reduction of viral replication and/or an increase in local infiltration of innate immune cells at the site of infection [33].

Basophils, among immune system players, are currently under investigation for their role in COVID-19 pathogenesis. At the beginning of the 20th century, Paul Ehrlich first described the presence of short-life peripheral blood cells presenting with a cytoplasm rich in basophilic granules. These cells were named basophils. Their resemblance with mast cells, not only because of the presence of basophilic granules in the cytoplasm but also for the expression on their surface of high-affinity IgE receptor, led researchers to consider these cells as minor precursors of tissue-resident mast cells. This theory remained valid until basophils were found to be one of the principal inducers of Th2 responses through IL-4 production, gaining a crucial role upon allergic and anti-parasitic protective immunity [34,35].

Indeed, basophils have crucial roles not only in the development of acute and chronic allergic responses through high-affinity IgE receptors (FcεRI), but also they demonstrated that they have protective immunity against ecto- and endoparasites [36,37].

Moreover, it is now well established that basophils can function as antigen-presenting cells (APCs). Indeed, basophils express MHC class II and costimulatory molecules such as CD80 and CD86 [38]. Therefore, they can induce Th2 cell differentiation through their functions as both APCs and IL-4 producers. During recent years, it was also noticed that basophils can bind antigens on their surface and boost humoral immune responses. In their study conducted on immunized mice to sepsis induced by Streptococcus pneumoniae, Denzel et al. showed that a depletion of basophils resulted in lower humoral memory response and greater infection susceptibility. Moreover, adoptive transfer of antigen-reactive basophils improved specific antibody production and B cell proliferation [39]. Another mechanism of B cells’ role upon adaptive immunity regarding humoral amplification was proposed by Kawakami. Indeed, it was postulated that after secondary immunization, basophils expressing FcεRI capture antigens through antigen-specific IgE previously produced by B cells after primary immunization. Activated basophils then secrete a panel of mediators, including IL-4 and IL-6, and express cell surface receptors, including CD40L. T cells switch to TH2 phenotype and induce amplified B cell response and antibody production in the presence of basophils. Basophil-derived IL-6 is critical, and IL-4 and cell contact (through CD40L-CD40 interactions) supports this process [4,40].

More recently, basophils were proven to also be involved in chronic inflammatory disorders through Th17 and Th17/Th1 cytokine expression. In their study, Wakahara et al. found a significant expression of basophils in lung and colon inflamed mucosa specimens, thus suggesting a role of these cells upon inflammatory disorders. They also demonstrated that circulating basophils may increase effector memory Th17 responses through ERK1/2 signaling pathway and partially via H2 and H4 histamine receptors [5,41].

A narrative review of the most significant research on this topic is described.

## 2. Search Strategy

We performed a strategic search through three different databases (PubMed, MedLine and Cochrane Medical Library) to find the studies in which basophil, mast cells, and COVID-19 disease were cited. Keywords used for the search were “COVID-19”, “SARS-CoV-2”, “basophil”, and “mast cell”.

Through this search, we found 31 manuscripts. By analyzing the abstract and the text for each of them, we selected only the papers that presented a study. The excluded papers were reviews, letters, and opinion papers. The included papers were 11 in total, 10 regarding basophils and 1 related to mast cells (Table 1).

## 3. Results

### 3.1. Basophils and COVID-19 Disease

On the basis of what has been previously exposed, we can assume an active role of basophils upon immune response to SARS-CoV-2. Indeed, according to previous studies on viral infections, basophil depletion might impair the efficacy of IgG-responses to SARS-CoV-2. Considering a comprehensive point of view, basophils could enhance a coordinate adaptive immune response to SARS-CoV-2 that could be suppressed by the hyperinflammatory reaction during the acute phase of COVID-19. Further investigation will be required to understand the mechanisms of basophils in modulating humoral responses to SARS-CoV-2. Interestingly, as the expression of these cells seems to be lower during acute disease, it could be worth determining the grade of this depletion as an important determinant of the antibody response to the virus [42].

In the study from Wuhan No. 1 Hospital [43], involving 59 male and 68 female Chinese patients, a reduction in basophil count was present in 13.39% of enrolled patients. Moreover, authors noted that the reduction in basophils was present in the first three days of hospitalization and was restored to normal shortly.

Fátima Conceição-Silva et al. [44] have shown that neutrophils, macrophages, lymphocytes, eosinophils, basophils, and mast cells can produce extracellular traps (ET), even if the modalities are still not completely known. Patients with severe cases of COVID-19 are predisposed to thrombosis in which ETs produced by neutrophils may participate. Contrary to neutrophils, ETs produced by basophils have a protective role against some infections with bactericidal and antifungal activity. A similar activity can also be hypothesized during COVID-19.

Our search shows that there is a tendency toward basopenia in COVID-19 patients.

A multidimensional analysis performed on laboratory parameters and diagnostic test of 178.887 Brazilian individuals, of whom 33.266 resulted in being positive for SARS-CoV 2 [45]. A case–control study [46] involving 74 COVID-19 patients and 228 non-COVID-19 patients, showed a significant reduction of basophil levels in COVID-19 patients, a result more evident in men older than 25 years of age in the Brazilian study.

A similar result was also found in a retrospective study on 120 COVID-19 patients, 100 influenza patients, and 61 healthy controls: basophils were lower both in the COVID-19 group and in the influenza group as compared to controls [47].

To further support these findings, researchers conducted an observational, multicentric study [48] that compared levels of complete blood count and granulocytes subsets with cytofluorimetric analysis in COVID-19 patients and healthy blood donors as controls. The authors showed a significant decrease in basophil levels in COVID-19 patients when compared to controls. Similar findings came from a retrospective study [49] on 548 patients diagnosed with COVID-19 disease performed by Chen et al., in which a difference between on admission and end-hospitalization levels of basophils greater than 0.02 × 109/L (HR, 2.73; 95% CI, 1.5–6.47) represented a risk factor for fatal outcome, thus suggesting that the less is the basophil count on admission the poorer is the outcome of the patient.

Analyzing severe cases of COVID-19 disease, in contrast with other low respiratory tract infections and excluding potentially confounding factors such as atopy and use of antihistamine drugs, Laing et al. [50] showed a dramatic depletion of plasmacytoid dendritic cells and basophils.

A similar lower percentage of basophils in the white blood cell count was found by Qin et al. [51] in an observational study involving 452 patients in severe patients compared to non-severe cases (0.1 vs. 0.2%; *p* = 0.015).

Further strength to these results is carried by the study of Sun Y. et al. [52] that evaluated the causal association between the different white blood cells and the COVID-19 susceptibility and severity by performing two-sample bidirectional Mendelian randomization analyses from the largest and most recent genome-wide association studies. Considering both severe COVID-19 disease and hospitalization due to COVID-19 disease as outcomes, the authors found an inverse association with low basophil count and low basophil percentage on white blood cell count ((OR = 0.75, CI: 0.60–0.95, *p* = 0.015), (OR = 0.70, CI: 0.54–0.92, *p* = 0.011)) and ((OR = 0.83, CI: 0.71–0.97, *p* = 0.020), (OR = 0.78, CI: 0.65–0.93, *p* = 0.005), respectively). The authors suggested a possible causal role of the reduced basophil count in increasing the risk of severe COVID-19 disease, potentially due to an insufficient innate immune response to SARS-CoV-2. No associations were found with COVID-19 susceptibility among white blood cells.

Contrary to what was observed for basophils, an histopathological study conducted on 6 SARS-CoV-2-confirmed patients compared to 10 H1N1-infected patients and a control group of 10 patients who died for neoplastic or cardiovascular diseases [53], showed a striking increase in the number of mast cells in lung of COVID-19 patients. Specifically, mast cells, even in the degranulated form, were more frequently localized in the perivascular spaces between the alveolar sacs and terminal bronchioles and in the alveolar septa, close to the alveolar capillaries. Authors suggested that mast cells may play an important role in triggering the systemic cytokine storm associated with severe COVID-19 because of their production of various mediators, including IL-4 and IL-6, two cytokines involved in COVID-19 disease pathogenesis [54].

As previously described, when SARS-CoV-2 infects the host, the host is firstly attacked by innate immune cells, including mast cells. The latter are well expressed by nasal epithelial and lung cells and their activation may be responsible of hyperinflammatory syndrome [55,56].

Mast cells are ubiquitous in the body, and they are involved in several conditions including viral infections, systemic inflammatory diseases, asthma, neuroinflammatory diseases, traumatic brain injury, stroke, and several stress disorders [44,56].

Hence, these cells proved to be very heterogeneous, as they may differ in ultrastructure, morphology, mediator content, receptor expression, and responses to various stimuli, thus explaining the different effect they may cause, protective or damaging. In fact, mast cell cytoplasm is enriched of histamine, proteases, heparin, chondroitin sulfate, and proinflammatory and anti-inflammatory cytokines/chemokines filled into granules or de novo produced upon activation, which are released in response to stimuli [57,58,59].

While physiologically these molecules are useful to defeat viral and microbial threats, they can also mediate the inflammatory damage typical of asthma and allergic reactions. Through the same mechanism, SARS-CoV-2 may exploit the same effect of mast cells, inducing an uncontrolled inflammation activation mediated by the release of histamines, proteases, cytokines, chemokines, and arachidonic acid compounds such as prostaglandin D2 and leukotrienes [60,61,62]. Indeed, current reports highlighted that COVID-19 can activate mast cells through TLRs and contribute to pulmonary inflammation and fibrosis [63,64].

**Figure 1:**
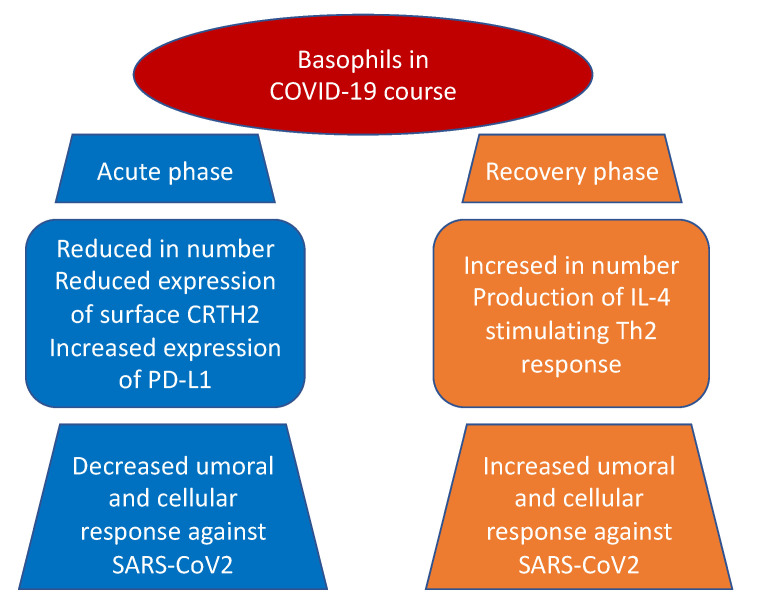
Basophils in COVID-19 pathogenesis.

It also emerged, both in basophils and eosinophils, a reduction in the expression of surface CRTH2, a receptor for prostaglandin D2, and a significant increase in programmed cell death ligand 1 (PD-L1) in the severe form of the disease in contrast with mild group. Interestingly, both WHO and SOFA scores correlated with these two findings: positively with PD-L1 and negatively with CRTH2, respectively (Figure 1). The authors suggested that one possible mechanism, shared by other viruses as an escape mechanism, may be the increase of immune checkpoint levels, which usually prevent immune-driven diseases after immune response in tissue in order to avoid the clearance of viral particles during the infection [65,66]. Moreover, the downregulation of basophil and eosinophil CRTH2, an important activator of T helper 2 polarized response, induces speculation about a possible inhibition of this cell subset by SARS-CoV-2. The authors concluded that this latter mechanism, which is present in allergy and hyper-eosinophilic asthma, can possibly be responsible for the protective effect conferred against SARS-CoV-2 infection and severity by these conditions [67,68].

Interestingly and in line with previous mentioned results, the study performed by Rodriguez et al. [42] showed a progressive increase in basophil and eosinophil levels from acute to recovery phase of the disease. They further demonstrated that basophil count rise was correlated to the immunoglobulin G response against SARS-CoV-2. Because of basophils are known to be able to bind antigens on their surface and improve humoral response through the production of IL-4 and/or IL-6, although levels of the latter were found to be inversely associated with specific antibodies, both authors concluded the presence of another mechanism, maybe related to IL-4 production, that may coordinate adaptive and humoral immune response to SARS-CoV-2. In fact, previous studies in viral infection showed similar results, with basophil level associated with humoral response [39] and with IL-4 known to enhance B cell activity against infection [40].

Moreover, basophil count was inversely correlated with IP-10, an IFN-γ-stimulated chemokine known to present chemo-attractive properties for monocytes, macrophages, T cells, NK cells, and dendritic cells [69,70]. The authors suggested that the CD68-positive infiltrate revealed by COVID-19 patient postmortem analysis [71] might explain the circulating loss of these cell populations, with basophils involved in tissue repair [72] and regulation of the coagulation process [73,74].

### 3.2. Mast Cell and COVID-19 Disease

**Figure 2:**
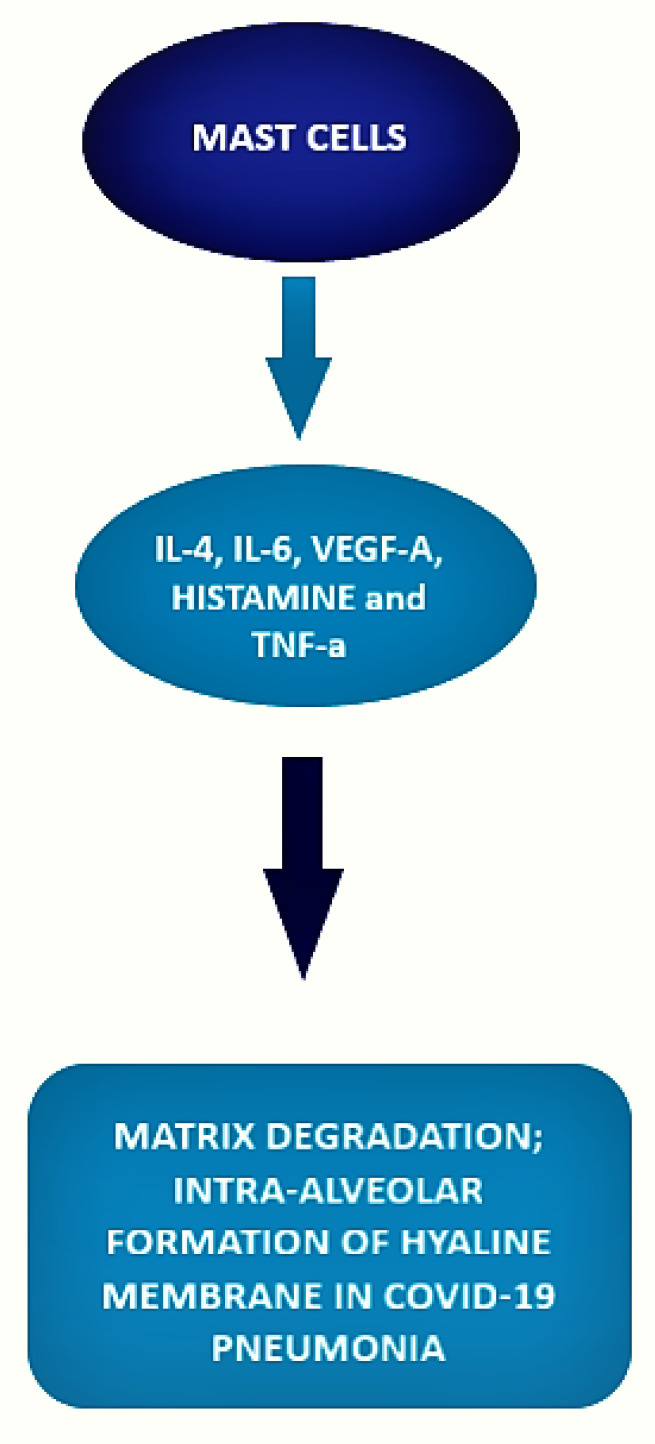
Role of mast cells in COVID-19.

The local inflammatory response in the lung observed in SARS-CoV-2-infected patients is characterized by a complex network of activated inflammatory innate immune cells, fibroblasts, endothelial cells, and bronchial epithelial cells. Bronchial epithelial cells and fibroblasts activated by SARS-CoV-2 cause upregulation of pro-inflammatory cytokines and induction of differentiation of mast cells that release histamine, proteases, cytokines, chemokines, and arachidonic acid compounds, such as prostaglandin D2 and leukotrienes, all of which are involved in the inflammatory network. Histamine is and is released into the vessels after cell stimulation. Histamine, stored endogenously within the secretory granules of mast cells, is involved in the increased expression of chemokine IL-8 and cytokine IL-6, thus favoring the hyperinflammation in the lung. Therefore, in the context of COVID-19 cytokine storm and severe disease, mast cells may act negative factors (Figure 2) as productors of histamine that induce microvascular leakage, proteases, and IL-6 that can degrade matrix, thus favoring intra-alveolar formation of the hyaline membrane and perpetuating inflammation, angiogenic factors and pro-coagulative factors, respectively, that may trigger immune thrombosis [75,76].

Interestingly, in this study, an increased number of IL-4-expressing cells were found in the alveolar septa of severe COVID-19 patients. This finding supports the above-mentioned results of Chen et al. [49] and Rodriguez et al. [42] regarding a possible mechanism that involves IL-4 in the pathogenesis and recovery phase of the disease. IL-4 is released by Th2 lymphocytes, mast cells, basophils, eosinophils, and innate lymphoid cells, thus representing a possible link between basophils and mast cells in the disease.

## 4. Discussion

Basophils and mast cells are important components of innate immune system and promoters of type 2 immune responses that protect primarily against parasitic infection and act in allergic reactions, especially anaphylaxis [54,75,76,77], but that may also play a role in viral infection [40,46,78,79,80,81].

Interestingly, our search demonstrates that both basophils and mast cells are probably involved in the pathogenesis of COVID-19 disease: the former may exert a protective role and the latter may play a key role in the pathogenesis. Indeed, basophil count and/or percentage seems to be reduced in COVID-19 patients as compared to controls [43,45,46,63,64,79], and this is the case in severe COVID-19 disease as compared to mild/moderate disease [49,50,51]. As mentioned in the introduction, basophils can act synergically with dendritic cells as antigen-presenting cells to CD4+ T cells and promote B cell response through induction of Th2 cell differentiation [39,40,82,83,84,85,86,87]. Concerning SARS-CoV 2 infection, it is likely that basophils play an important role in promoting antibody response thanks to their capability to release IL-4 [42]. IL-4 may represent a central cytokine in the pathogenesis of severe COVID-19 pneumonia. A recent post-mortem analysis of lung biopsy samples of patients who died of SARS-CoV-2 showed significantly higher IL-4 tissue expression in COVID-19 patients compared both to H1N1 and control patients. Moreover, SARS-CoV-2 seems to promote lung damage through a higher participation of the Th2 response [86].

Because of IL-4 may be secreted both by basophils and mast cells and, as demonstrated by Motta J. et al. [53], IL-4-expressing cells are increased in lung biopsy from COVID-19 patients as well as resident mast cells, this cytokine may possibly be the link between these two different cell populations. IL-4 can impair endothelial barrier by remodeling, upregulation of the expression of vascular cell adhesion molecule-1 (VCAM-1), and monocyte chemotactic protein-1, inducing hyperpermeability and causing microvascular leakage [87].

In this context, mast cells may take part in the pro-coagulative status typical of COVID-19 patients by releasing pro-angiogenic VEGF-A, histamine, and tumor necrosis factor-α, thus leading to endothelial activation in inflamed alveolar septa and subsequent fibrin formation through activation of both extrinsic and intrinsic coagulation pathways [88,89].

In conclusion, more evidence is needed to further improve our knowledge about basophil and mast cells in viral infections. Studies on SARS-CoV 2 infection and involved cell populations and cytokines may represent a chance to better understand physiologic and physio-pathologic mechanisms of viral infections and human immune response that may, in the future, lead us to new therapies and approaches.

## Figures and Tables

**Table 1:** Summary of the literature with the main findings of the basophil and mast cell involvement in COVID-19 pathogenesis.

Reference and Type of Study	No. of Patients	Main Results
Rodriguez et al. [42] Systems-level blood immune-monitoring study	37 patients	Basophils were depleted during acute disease but increase during recovery. Moreover, levels of basophils were significantly correlated with the titers of IgG antibodies to SARS-CoV-2.
Mao et al. [43] Observational study	127 patients admitted at Wuhan No. 1 Hospital	Basophil count was low in 17 (13.39%) patients during the first three days of hospitalization and returned to normal levels shortly after.
Fátima Conceição-Silva et al. [44] Review discusses the presence of ETs		ETs produced by neutrophils were associated with severity in SARS-CoV-2 infection favoring thrombosis. ET produced by basophils, which have bacteral killing and antifungal activity, could have a protective role during COVID-19 infection.
Ten-Caten et al. [45] A multidimensional analysis	178,887 Brazilian individuals, of which there were 33,266 positives for SARS-CoV-2	Lower counts of platelets, basophils, lymphocytes, and eosinophils were observed in COVID-19 cases compared with controls in both males and females.
Alnor et al. [46] Case–control study	74 COVID-19-positive and 228 COVID-19-negative patients.	COVID-19 patients presented significant lower values for all white blood cells, including basophil count, in comparison with non-COVID-19 patients.
Kazancioglu et al. [47] Retrospective case–control study	120 COVID-19 patients, 100 influenza patients, and 61 healthy controls	Lower basophil count was found both in COVID-19 and influenza patients compared to healthy controls.
Vitte et al. [48] Observational, prospective, multicentric, case–control study	26 patients	Low number of basophils were detected in COVID-19 patients as compared with healthy donors. Both CRTH2 (CD294), a receptor for prostaglandin D2, and CD11b expression were decreased at the surface of basophils. SARS-CoV-2 infection was associated with inhibition of T helper 2 polarized immune responses and a decreased chemotaxis of CRTH2+ cells, through reduction of basophils, eosinophils, and CRTH2 itself.
Chen et al. [49] Retrospective study	548 patients with COVID-19	A difference of basophil levels between admission and end-hospitalization greater than 0.02 × 10^9^/L (HR, 2.73; 95% CI, 1.5–6.47) resulted a risk factor for fatal outcome.
Laing et al. [50] Observational study	63 patients	Basophils and dendritic cells are reduced in severe COVID-19. Basophil reduction correlate with elevated interferon-inducible protein-10.
Qin et al. [51] Observational study	452 patients admitted at Tongji Hospital	Severe COVID-19 patients had a lower percentage of basophils than non-severe ones (0.1 vs. 0.2%; *p* = 0.015).
Sun et al. [52] Mendelian randomization study	408,112 and 562,132 European subjectsfrom two different GWAS	Negative association between basophil count and basophil percentage of WBC are present in severe and hospitalized COVID-19 disease.
Motta et al. [53] Histopathological comparative control study	6 post-mortem covid patients, 10 post-mortem H1N1-infected patients, 10 patients who died for different reasons.	COVID-19 patients samples presented a significantly higher number of mast cells as compared con H1N1 patients and controls. In the context of a neutrophilic endothelitis, mast cells were more frequently localized in the perivascular spaces between the alveolar sacs and terminal bronchioles and in the alveolar septa, close to the alveolar capillaries.
Bahareh Hafezi et al. [54] cytokine response by mast cells during COVID-19.		MCs can respond to SARS-CoV-2 and accumulate in the lungs of patients with COVID-19, where they correlate with pulmonary edema, inflammation, and thrombosis.
